# Reliability of Load-Velocity Profiling in Front Crawl Swimming

**DOI:** 10.3389/fphys.2020.574306

**Published:** 2020-09-23

**Authors:** Bjørn Harald Olstad, Tomohiro Gonjo, Nikolai Njøs, Kathrin Abächerli, Ola Eriksrud

**Affiliations:** ^1^Department of Physical Performance, Norwegian School of Sport Sciences, Oslo, Norway; ^2^Department of Health Professions, Bern University of Applied Sciences, Bern, Switzerland

**Keywords:** accuracy, semi-tethered, strength, performance, testing, ICC, Bland-Altmann analysis, multiple trial method

## Abstract

The purposes of this study were to establish test-retest reliability of calculating load-velocity profiles in front crawl swimming using five and three different external loads, and if outcome results were comparable between calculation methods for monitoring performance over time. Fifteen swimmers at either national or international competition level (seven females and eight males) participated in this study. The subjects performed 25 m of semi-tethered swimming with maximal effort with five progressive loads (females 1, 2, 3, 4, and 5 kg and males 1, 3, 5, 7, and 9 kg) as well as 50 m maximal front crawl on 2 different days. The mean velocity during three stroke cycles in mid-pool was calculated and plotted as a function of the external load. Relationship between the load and velocity was expressed by a linear regression line and established for each swimmer. The intercepts between the axes of the plot and the established regression line were defined as theoretical maximum velocity (V_0_) and load (L_0_). In addition, L_0_ was also expressed as a percentage of body mass (rL_0_). The coefficient of determination (R^2^) and the slope (S_lv_) of the linear load-velocity relationship were calculated. The intra-class correlation coefficient (ICC) showed excellent agreement (ICC ≥0.902) for all variables. The coefficient of variation was ≤3.14% and typical error was rated as “good” in all variables. A difference was found between day 1 and 2 in V_0_ for three- and five-load calculations and for 50 m front crawl time (*p* < 0.05). No difference was found between the load-velocity profile outcomes variables compared between the three- and five-trial protocols on neither day 1 nor 2. The Bland-Altman plots showed a small bias across all resistance conditions for five loads, L_0_: 0.04 kg, rL_0_: 0.13%, V_0_: −0.03 m/s, and S_lv_: 0.003 −m/s/kg and for three loads, L_0_: −0.24 kg, rL_0_: −0.27%, V_0_: −0.04 m/s, S_lv_: 0.002 −m/s/kg. In conclusion, the load-velocity profile for front crawl swimming can be calculated with high reliability from both five and three external loads and comparable results in outcome variables were established. These methods can be used to monitor performance parameters over time, and to investigate and compare swimmers’ velocity and strength capabilities to allow for individualized training prescription to improve performance.

## Introduction

Force-velocity profiles of locomotive patterns, such as sprint running, have been used to understand how these two performance indicators interact (Cross et al., 2018a; Jiménez-Reyes et al., 2019, 2020). Such an approach can also be useful in sprint swimming. However, even though the net-force in the swimming direction (i.e., the sum of the propulsive and resistive forces) can be obtained by the inverse-dynamics approach (Sanders et al., 2015), measuring the propulsive and resistive forces in the water separately is complex due to unsteady manners of the water flow around swimmer’s body (Samson et al., 2018).

One way of overcoming the complexity is to apply a fully tethered swimming approach, in which a swimmer is attached to an inelastic cord - the other end of which is attached to a fixed force transducer (Amaro et al., 2014, 2017). With this method, a tested swimmer does not move forward; thereby, the measured force can be interpreted as the force the swimmer produced for a propulsive purpose. However, since the swimmer do not produce any forward velocity, fully tethered swimming is not applicable for establishing the force-velocity profile.

An alternative method is a semi-tethered swimming approach, in which a swimmer is required to swim with a known external load applied by a pully system (Dominguez-Castells et al., 2013; Cuenca-Fernández et al., 2020), a floating object (Kolmogorov and Duplishcheva, 1992; Morais et al., 2020), or a resistance device (Gonjo et al., 2020). This method allows researchers to conduct a similar assessment as bespoken on-land force-velocity studies. Consequently, employing the multiple trial method using different external resistive loads instead of force (Cross et al., 2018a,b) with a series of semi-tethered swim tests is an alternative to force-velocity testing (Gonjo et al., 2020). Outcome variables such as maximum load at zero velocity (L_0_), maximum velocity at zero load (V_0_), steepness of the linear slope for the load-velocity relationship (S_lv_) can be used to understand (Cross et al., 2017b), monitor (Jiménez-Reyes et al., 2019, 2020), and prescribe training programs (Cross et al., 2018a).

Despite the potential, semi-tethered swimming approaches have mostly been applied to assess the net swimming power that is the product of the net-force and the swimming velocity (Shionoya et al., 1999; Dominguez-Castells et al., 2013; Kimura et al., 2013), and there is only one study employing the method to investigate swimming load-velocity profiling (Gonjo et al., 2020). Furthermore, the reliability of the method has not been investigated. It has been reported that fully-tethered swimming is highly reliable to assess the maximum and mean tether force (Amaro et al., 2014), which implies that swimmers can produce stable swimming force and motion in a tethered condition. However, given the differences between fully- and semi-tethered swimming approaches such as single and multiple trials and potential technical changes due to distinct relative flow velocity around the body, it is still unclear whether or not a semi-tethered swimming testing is also a reliable method. Therefore, establishing the reliability of swimming load-velocity profiling with a semi-tethered swimming approach is essential to utilize the method as a test to understand individual and group level performance, monitoring performance over time, and prescribe training programs as done in sprint running (Cross et al., 2017b, 2018a; Jiménez-Reyes et al., 2019).

Ensuring the reliability would also expand possibilities for researchers to conduct a biomechanical or physiological investigation with the method. The number of trials to be used should also be considered carefully as it will decrease time of testing and minimize the effect of fatigue that could lead to an overestimation of V_0_ (Gonjo et al., 2020), especially when performing a heavy load trial (Driss and Vandewalle, 2013). However, it is unclear if a different number of trials affect the reliability of the measurement. The purpose of this study was therefore to explore test-retest reliability of load-velocity profile outcome variables in front crawl swimming and to compare calculations using five and three different loads.

## Materials and Methods

### Subjects

Sixteen swimmers at either national or international competition level [mean ± standard deviation (SD): 17.3 ± 1.5 y, 178.0 ± 8.8 cm, 68.9 ± 7.7 kg, 690 ± 77.7 FINA Points and 50 m front crawl personal best time 26.1 ± 1.9 s] including eight females (mean ± *SD*: 17.6 ± 1.2 y, 171.2 ± 6.1 cm, 64.8 ± 7.2 kg, 689.6 ± 91.4 FINA Points and 50 m front crawl personal best time 27.8 ± 0.9 s) and eight males (mean ± SD: 17.0 ± 1.8 y, 184.9 ± 4.6 cm, 73.0 ± 6.4 kg, 690.4 ± 67.8 FINA Points and 50 m front crawl personal best time 24.5 ± 1.0 s) volunteered for the study. The highest number of achieved Fédération internationale de natation (FINA) points for each swimmer regardless of distance and stroke within the last year was used. The swimmers were recruited from a local swimming performance high school and the junior and youth national team. Inclusion criteria were set to minimum 5 years of participation in competitive swimming, training at least seven times and 15 h per week, competing at the national level in any stroke or distance and no current medical conditions. No subjects met any of the exclusion criteria: heart disease (high blood pressure and high cholesterol), diabetes, vertigo, balance disorders, sick, or injured during the week prior to testing from past and current medical conditions. However, one subject was excluded during the data analysis for not containing a 1 kg trial on day 1 of the experiment, which resulted in a total analyzed subject number of fifteen (seven females and eight males).

The study was approved by the local Ethical committee and the National Data Protection Agency for Research in accordance with the Declaration of Helsinki. Prior to participation, all subjects completed a questionnaire including details on training activity, injuries, sicknesses and family history. The subjects were given detailed verbal and written explanation of the purpose, procedures and risks associated with participation. No nutritional recommendations were imposed on the subjects outside of their daily routines and they were instructed to abstain hard physical training for the last 24 h prior to testing. Subjects or the legal guardian (for minors) provided written informed consent prior to participation.

### Procedures

The experiment was performed in a 25 m indoor swimming pool with water and air temperature of 27 and 28°C, respectively.

The subjects first performed their individual standardized warm-up procedure on land and in water as they do before a competition for ∼45 min. After warm-up, the subjects performed a 50 m front crawl with maximal effort, in which the finishing time was recorded by an automatic timing system (Omega, Bienne, Switzerland). Following a 10–20 min rest, subjects were required to perform five 25 m front crawl sprints with maximal effort with different loads. The loads for the female swimmers were 1, 2, 3, 4, and 5 kg, while the loads for the male swimmers were 1, 3, 5, 7, and 9 kg (assessed in ascending order). These external loads were decided based on pilot testing to avoid large decelerations throughout the heaviest load (Gonjo et al., 2020) and to impose a reduction in velocity (%V_dec_) compared with the lightest load to be categorized as heavy resistance (>30%V_dec_) (Petrakos et al., 2016). Each sprint was initiated from a push-off start followed by surface swimming at the 5 m mark. In order to attempt total recovery between each sprint, recovery time was ∼6 min (Hancock et al., 2015). During the experiment the swimmers were not blinded from each other or excluded from interacting with each other as this would not be feasible in future studies or assessments of swimmers. To provide data for the reliability calculations, the same procedures were undertaken 1–5 days later at the same time of the day.

A portable robotic resistance device 1080 Sprint (1080 Motion AB, Lidingö, Sweden) featuring a servo motor (200 RPM OMRON G5 Series Motor, OMRON Corporation, Kyoto, Japan) was used to measure the swimming velocity and to add an external load on swimmers. The device was positioned on the starting block 1 m above the water surface to minimize the disruption of swimming technique (especially kicking) by a fiber cord connecting the device and swimmer (Amaro et al., 2017). Subjects were instructed to wear a S11875BLTa swim belt (NZ Manufacturing, OH, United States) around their pelvis to connect the cord. An illustration of the experimental set-up can be found in Gonjo et al. (2020). The settings for the 1080 Sprint were; isotonic resistance mode, gear 1, eccentric and concentric velocity of 0.05 and 14 m/s, and load parameters (kg) presented previously. Data was acquired with a sampling frequency of 333 Hz from the 5.0 to the 20.4 m mark for each 25 m trial.

Data was imported to MATLAB R2019b (MathWorks, Natick, MA, United States) as text files for further processing. Three stroke cycles around the middle of the pool that generated the highest R^2^ value were selected to calculate the parameters of the load-velocity profile. The window was chosen to avoid the impulse from the wall push-off and the speed decrease at the end of each trial (Dominguez-Castells et al., 2013). Since the cord that was used for the velocity measurement was not aligned with the swimming direction, the following equation was used to obtain the horizontal velocity component (Gonjo et al., 2020).

$$V_{a\,d\,j}=V\cdot \cos\left[\sin^{-1}\left(1.00/\text{Lw}\right)\right]$$

V and V_adj_ are the measured velocity by the machine and the horizontal component of the velocity, respectively. 1.00 is the height (m) above the water surface where the cord is stretched out from the device, and L_w_ is the length of the cord (m) from the device to the swimmer at each sampling time. The mean V_adj_ from the three stroke cycles was plotted as a function of the corresponding external load (kg). A linear regression line was established for each subject (Dominguez-Castells and Arellano, 2012) based on the load-velocity plot for three and five trials. For all calculation modelings using three different loads, female swimmers had 1, 3, and 5 kg, while males had 1, 5, and 9 kg. V_0_ and L_0_ were predicted from the regression line by obtaining the intercepts of the line with the vertical and horizontal axes, respectively. Coefficient of determination (R^2^) and S_lv_ (the steepness of the linear slope for the load-velocity relationship, computed as S_lv_ = –V_0_/L_0_) were calculated. L_0_ was also expressed as a percentage of body mass (rL_0_).

### Statistical Analyses

The Statistical Package for Social Sciences (SPSS) version 24.0 (IBM Corp, Armonk, NY, United States) and Excel for Microsoft 365 (Microsoft Corp, Redmond, WA, United States) were used for all statistical computations.

The Shapiro-Wilk test was used to check the normal distribution of the data, which was met for all variables. Descriptive analysis of the load-velocity profile parameters (L_0_, V_0_, rL_0_, R^2^, and S_lv_) are reported as mean and standard deviation. Test-retest reliability of each parameter was assessed using intra-class correlation (ICC) with a two-way random single-measure model (Bartko, 1966), absolute error (AE), typical error (TE), coefficient of variation (CV), standard error of measurement (SEM), and minimal detectable change based on a 95% confidence interval (MDC). The ICC was classified as <0.5: poor, 0.5–0.75: moderate, 0.75–0.9: good, and >0.9 excellent agreement (Koo and Li, 2016). A SEM smaller than, similar to or larger than the MDC was rated as “good,” “ok,” or “marginal,” respectively (Buchheit et al., 2011). It is important to assess the change in the mean other than within-participant variation and retest correlation (Hopkins, 2000). Therefore, a paired sample *t*-test was used to compare the outcome parameters between the two sessions as well as between the three and five loads calculations. Because females and males were assessed with different external loads, a Mann-Whitney *U-*test was used to compare their response in %V_dec_ for the protocols between the lightest and heaviest load. The level of statistical significance was set at *p* < 0.05. Furthermore, Bland-Altman analysis was used to display the within-subject variation as well as the systematic change between the sessions: bias (mean difference), standard deviation (SD) and upper and lower limits of agreement (defined as MD ± 1.96 × SD) were calculated (Bland and Altman, 1986).

## Results

Test-retest reliability for load-velocity profiling parameters from five and three different load conditions are displayed in Tables 1, 2, respectively. The ICC showed excellent agreement for all variables (L_0_, rL_0_, V_0_, and S_lv_) with both five and three loads calculations. CV was 3.14% for S_lv_ with five loads and < 3% for all remaining variables with five and three loads calculations. SEM was rated as “good” in all variables when compared to MDC. A significant difference was found between day 1 and 2 in V_0_ for both three- and five-loads calculations and for 50 m front crawl time (*p* < 0.05). No difference was found between the load-velocity profile outcome variables compared between the three and five loads calculations on either day 1 or 2 (*p* > 0.05). There was no difference between the protocols for females and males in terms of %V_dec_ between the lightest and heaviest load, day 1 (females 40.6 ± 7.8% and males 55.5 ± 16.9%, *p* = 0.09) and day 2 (females 41.7 ± 8.9% and males 53.0 ± 14.3%, *p* = 0.12).

**TABLE 1:** Mean variables from the load-velocity profiling (five different loads).

Variable	Test (*mean* ± *SD*)	Retest (*mean* ± *SD*)	*p*-value	AE	TE	CV (%)	ICC	CI_lower95%_	CI_upper95%_	SEM	MDC
L_0_ (kg)	14.16 ± 4.57	14.20 ± 4.51	0.0872	0.71	0.35	2.53	0.980	0.942	0.993	0.65	1.80
rL_0_ (%)	20.31 ± 4.81	20.45 ± 4.94	0.0695	1.03	0.52	2.53	0.966	0.904	0.989	0.90	2.49
V_0_ (m/s)	1.73 ± 0.13	1.69 ± 0.16	0.012	0.04	0.02	1.39	0.923	0.677	0.977	0.03	0.07
S_lv_ (−m/s/kg)	−0.13 ± 0.03	−0.13 ± 0.03	0.165	0.01	0.00	3.14	0.948	0.855	0.982	0.01	0.02
R^2^	0.99 ± 0.01	0.99 ± 0.01									
50 m FC time (s)	26.30 ± 2.10	26.54 ± 2.35	0.039	0.36	0.18	0.66	0.978	0.920	0.993	0.23	0.64

*L_0_, estimated maximum load from the load-velocity slope; rL_0_, estimated maximum load as a percentage of body mass; V_0_, estimated maximum velocity from the load-velocity slope; S_*lv*_, teepness of load-velocity regression line; R^2^, coefficient of determination of the load-velocity regression line; FC, front crawl; SD, standard deviation; AE, absolute error; TE, typical error; CV, coefficient of variation; ICC, intra-class correlation coefficient; CI_*upper95%*_, upper bound of 95% confidential interval of Mean; CI_*lower95%*_, lower bound of 95% confidential interval of Mean; SEM, standard error of measurement; MDC, minimal detectable change.*

**TABLE 2:** Mean variables from the load-velocity profiling (three different loads).

Variable	Test (mean ± *SD*)	Retest (mean ± *SD*)	*p*-value	AE	TE	CV (%)	ICC	CI_lower95%_	CI_upper95%_	SEM	MDC
L_0_ (kg)	14.22 ± 4.63	13.97 ± 4.23	0.289	0.62	0.31	2.16	0.981	0.946	0.994	0.53	1.46
rL_0_ (%)	20.39 ± 4.85	20.12 ± 4.50	0.361	0.84	0.42	2.11	0.973	0.923	0.991	0.73	2.01
V_0_ (m/s)	1.72 ± 0.13	1.68 ± 0.15	0.010	0.04	0.02	1.38	0.902	0.589	0.971	0.03	0.09
S_lv_ (−m/s/kg)	−0.13 ± 0.03	−0.13 ± 0.03	0.456	0.01	0.00	2.59	0.962	0.893	0.987	0.01	0.02
*R*^2^	0.99 ± 0.01	0.99 ± 0.01									
50 m FC time (s)	26.30 ± 2.10	26.54 ± 2.35	0.0039	0.36	0.18	0.66	0.978	0.920	0.993	0.23	0.64

*L_0_, estimated maximum load from the load-velocity slope; rL_0_, estimated maximum load as a percentage of body mass; V_0_, estimated maximum velocity from the load-velocity slope; S_*lv*_, steepness of load-velocity regression line; R^2^, coefficient of determination of the load-velocity regression line; FC, front crawl; SD, standard deviation; AE, absolute error; TE, typical error; CV, coefficient of variation; ICC, intra-class correlation coefficient; CI_*upper95%*_, upper bound of 95% confidential interval of Mean; CI_*lower95%*_, lower bound of 95% confidential interval of Mean; SEM, standard error of measurement; MDC, minimal detectable change.*

**FIGURE 1:**
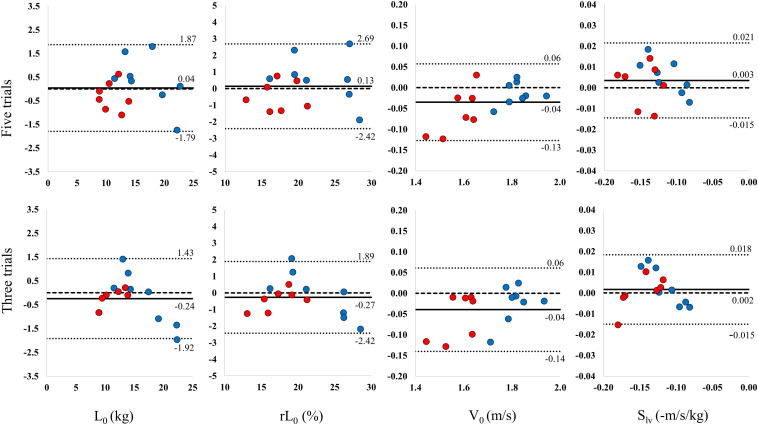
Bland-Altman plots of the difference between test and retest (*y*-axis) vs. mean of measurements (*x*-axis) of load-velocity profile parameters L_0_, rL_0_, V_0_, and S_lv_ with zero difference (dashed line), bias (thick line) and with lower and upper limits of agreement (dotted lines). The sample size for each Bland-Altman plot is *n* = 15 with seven female and eight male subjects marked with red and blue dots, respectively. L_0_, estimated maximum load from the load-velocity slope (kg); rL_0_, estimated maximum load as a percentage of body mass (%/kg); V_0_, estimated maximum velocity from the load-velocity slope (m/s); S_lv_, slope of load-velocity regression line (−m/s/kg).

Distribution of the load-velocity profile variables is presented in Figure 1. Biases (_diff_) were small across all resistance conditions for five loads (L_0_: 0.04 kg, rL_0_: 0.13%, V_0_: −0.04 m/s, and S_lv_: 0.003 −m/s/kg) and for the three loads conditions (L_0_: −0.24 kg, rL_0_: −0.27%, V_0_: −0.04 m/s, S_lv_: 0.002 −m/s/kg).

**FIGURE 2:**
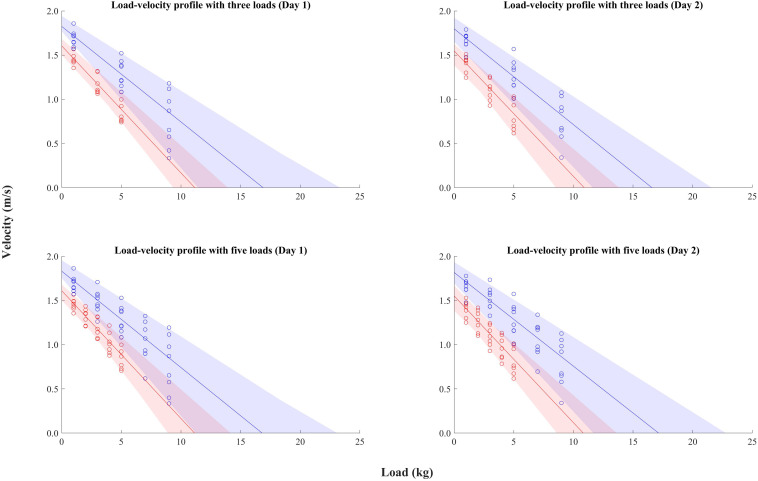
The average curve and range for the load-velocity profile of the 15 subjects. Seven female and eighet male subjects are marked in red and blue, respectively. Each dot represents the individual value for each subject.

The average curve and range for the load-velocity profile of the 15 subjects are presented in Figure 2. L_0_ mean were 16.8 and 17.1 kg (five loads) and 16.9 and 16.7 kg (three loads) for males, and 11.1 and 10.8 kg (five loads) and 11.2 and 10.9 kg (three loads) for females in day 1 and 2, respectively. V_0_ mean were 1.8 and 1.8 m/s (five loads) and 1.8 and 1.8 m/s (three loads) for males, and 1.6 and 1.6 m/s (five loads) and 1.6 and 1.5 m/s (three loads) for females in day 1 and 2, respectively.

## Discussion

The aim of this study was to determine the test-retest reliability of load-velocity profile outcome measurements derived from front crawl semi-tethered swimming with five and three different external loads. Overall, the method has excellent reliability for both the three and five trial approach and no difference was observed for any outcome measurements between the three and five load calculations.

The observed reliability of the load-velocity variables obtained in the present study are similar or better to those observed in multiple trial resisted sprints (Cross et al., 2017a; Cahill et al., 2019, 2020) and cycling (McCartney et al., 1983; Dore et al., 2003). The observed ICC values were excellent for both the three (range: 0.902–0.981) and five (range: 0.923–0.980) trial calculations, which are greater than those reported for multiple trials resisted sprinting (Cross et al., 2017a; Cahill et al., 2019, 2020). The observed CV range: for three (1.4–2.6) and for five (1.4–3.1) loads are comparable to what has been reported in resisted sprinting (Cross et al., 2017a; Cahill et al., 2019, 2020) and in cycling (Dore et al., 2003). Considering the marginal influence on reliability outcome variables from the calculations with five and three trial method, the three-trial method is sufficient to assess load velocity profiles of semi-tethered freestyle swimming.

The heaviest load (5 kg for women and 9 kg for men) gave a reduction in velocity compared with the lightest load (1 kg) by around 49% V_dec_, which is categorized as heavy resistance (>30%V_dec_) (Petrakos et al., 2016). In addition, there was no difference in %V_dec_ between females and males on either day 1 (*p* = 0.09) or day 2 (*p* = 0.12). One could argue that using heavier loads might be better for some subjects to ensure assigning trials with a load close to L_0_. However, the use of up to 5 or 9 kg load in the present study is justified by the average R^2^ values of the load-velocity profiles 0.99 for all conditions. They are comparable to findings from resisted sled sprints (0.99) with < 60%V_dec_ (Cross et al., 2017a), and other studies using multiple trial method on semi-tethered swimming (0.97–0.99) (Dominguez-Castells and Arellano, 2012; Gonjo et al., 2020). This shows a clear and robust linear relationship between the velocity and load parameters in semi-tethered swimming, indicating that more than three loads with extremely heavy loads are unnecessary. A reduction in the number of trials is important as it could minimize the effect of fatigue when predicting L_0_ and V_0_ (Driss and Vandewalle, 2013). For example, failing to perform with maximal swimming velocity at a heavy load due to fatigue would lead to a steeper S_lv_ than it should be, and therefore causes an overestimation of V_0_ (Gonjo et al., 2020).

Given the excellent ICC for both five and three load calculations (0.948 and 0.962, respectively) and CV (3.1 and 2.6%, respectively), S_lv_ can be used as an index of the individual balance between velocity and load (strength) capabilities of each swimmer. A steep S_lv_, expressed by a large negative value, would indicate that the swimmer is “velocity oriented,” and vice versa (Morin and Samozino, 2016). An example from the present study is two male swimmers, both with a V_0_ of 1.83 m/s. The L_0_ was 19.80 and 12.49 kg, generating a S_lv_ of −0.09 and −0.15, respectively. This indicates that the first swimmer would be load dominant while the second is velocity dominant. While there is currently no optimal load-velocity profile established for front crawl swimming as there is for ballistic movements (Samozino et al., 2012), this value could be utilized to identify swimmers who are velocity or load dominated and subsequently used to prescribe training programs to target an imbalance and thereby enhance swimming performance.

Despite the good and excellent reliability suggested by the analyzed variables, V_0_ was significantly lower (around 2%) in the second day compared with the first day for both five and three loads calculations. A systematic change in a test-retest investigation design can be due either to the participants (e.g., physical and psychological condition) or the setting of the equipment used. Several results of the current study imply that the bias was probably due to the participant rather than the testing equipment or setting; firstly, 50 m front crawl time was also significantly slower in the second than the first day, which means that swimmers’ condition was slightly worse in the second day; secondly, the systematic change was not observed in L_0_, rL_0_, and S_lv_ (*p* > 0.05), suggesting that the error was not due to the equipment setting or preparation since the three outcome measures are not entirely independent but related to each other (i.e., if there was a systematic error due to the equipment, there should have been at least two variables with a systematic change). Therefore, it should be noted that the reliability in V_0_ might have been underestimated because of the systematic bias. Nevertheless, even with the systematic bias, the test-retest error in V_0_ in the current study was similar to or better than on-land load-velocity profiling (Samozino et al., 2016; Cahill et al., 2019), which reinforces the reliability of swimming load-velocity profiling.

Investigating the load-velocity profile in swimmers with different distance specialities could be practically useful. Even though (Rodríguez and Mader, 2011) sprint and distance swimmers exhibit a similar motion in both sprint and distance paces (McCabe et al., 2011; McCabe and Sanders, 2012), long-distance swimmers are characterized by a greater percentage of Type I muscle fiber than sprint swimmers, suggesting less muscular power capabilities than sprint athletes (Gerard et al., 1986). Therefore, despite the similarity in the motion, it is probable that swimmers would exhibit a different load-velocity profile depending on their speciality due to the neuromuscular difference. It would also be of interest to compare load-velocity profile between competitive swimmers and triathletes or open water swimmers. Distance competitive swimmers, triathletes, and open water swimmers could all be categorized as endurance athletes; therefore, neuromuscular differences between those athletes might not be as evident as the difference between sprint and distance swimmers. However, contrary to the similar kinematics among competitive swimmers, competitive swimmers and triathletes show distinct kinematic characteristics (Millet et al., 2002), which might affect the load-velocity profile.

In all variables obtained in this study, V_0_, L_0_, rL_0_, and S_lv_ showed smaller SEM than MDC. This demonstrates lower error in the measurement compared to detecting an actual change in performance or in the parameter of interest. However, this study had a heterogeneous population including both women and men at different performance levels, and therefore MDC should be interpreted with caution. For example, the interpretation of MDC of 1.46 kg for L_0_ with three loads would be quite different for subjects scoring L_0_ of around 9 kg or over 23 kg. The MDC value in this study should foremost be understood as a variable used to assess the reliability, and further studies are necessary to establish MDC values to predict changes in performance for subjects of different genders, ages and performance levels. Therefore, another approach for detecting a change in performance for subjects with low L_0_ can be calculated using 1.5–2.0 times the TE (Hopkins, 2000). This approach would then yield a change in L_0_ between 0.47 and 0.62 kg (using three loads) to identify that a change in performance has occurred.

### Limitations

One limitation of the present study was a mixed-gender in the limited number of analyzed samples. As clearly seen in Figure 2, male and female swimmers showed different V_0_ and L_0_, implying that obtained results based on the absolute numerical outcomes (such as AE, TE, SEM, and MDC) might have been biased due to the gender difference. However, the current study also investigated relative test-retest error as CV, and the test-retest agreement was also checked using ICC. Despite the difference in the absolute numerical values between genders, these variables should support the reliability of the testing method if both males and females responded to the testing in a comparable manner. Given that no difference was observed between females and males in terms of %V_dec_ from the lightest to heaviest load trials, it is probable that swimmers responded to the prescribed loads similarly regardless of the gender. Therefore, despite the possibility of the gender effect on the results, it can still be concluded that the current study established the reliability of swimming load-velocity profiling method.

Devices that put a constant load on swimmers might not be available for all practitioners and there are some time requirements involved to properly secure and set-up the device on the starting block. Simpler alternatives could therefore be explored (e.g., parachutes together with a stopwatch). A critical matter for alternative methods would be to standardize the external loads and obtain accurate time measurements.

## Practical Applications

Load-velocity profiling with three different loads can be a practical and time efficient performance test that allows coaches and practitioners to investigate and compare velocity and strength capabilities of swimmers. The outcome parameters of a load-velocity profile can allow the prescription of individualized training for improving performance. This reliable performance test will also be of help to establish requirements for performance at different levels. Future research should therefore examine the load-velocity profile relationship with swimming performance for different strokes, distances and genders. It would also be of interest to compare load-velocity profile between competitive swimmers and triathletes or open water swimmers as well as for other swimming-based sports. Attempts should also be made for establishing optimal S_lv_’s to determine the preferred balance between velocity and strength capacities as well as training intervention.

## Conclusion

The load-velocity profile for front crawl swimming can be calculated with high reliability using both five and three different loads. This means that load-velocity profiling can be used to assess swimming specific strength and velocity capabilities related to performance over time. It enables practitioners to investigate and compare swimmers’ velocity and strength capabilities allowing individualized training prescriptions.

## Data Availability Statement

All datasets generated for this study are included in the article/Supplementary Material.

## Ethics Statement

Studies involving human subjects were reviewed and approved by the local ethical committee at the Norwegian School of Sport Sciences, reference number 47 - 060218-200318. The subjects provided their written informed consent to participate in this study.

## Author Contributions

All authors listed have made a substantial, direct and intellectual contribution to the work, and approved it for publication.

## Conflict of Interest

OE was a shareholder in 1080 Motion AB. The remaining authors declare that the research was conducted in the absence of any commercial or financial relationships that could be construed as a potential conflict of interest.
