# Supplementary material for: Reliability of Load-Velocity Profiling in Front Crawl Swimming
Source: Front Physiol. 2020 Sep 23;11:574306. doi: 10.3389/fphys.2020.574306 (PMC7538691; doi:10.3389/fphys.2020.574306)
Supplement: Supplementary file 1 [file Data_Sheet_1.pdf]

Supplement 3. The MATLAB script for processing the data.

Olstad BH, Gonjo T, Njøs N, Abächerli K and Eriksrud O (2020)
Reliability of Load-Velocity Profiling in Front Crawl Swimming.
Front. Physiol. 11:574306. doi: 10.3389/fphys.2020.574306

```
clear all
close all



Marker = {'o','x','s','d','p','+','^','v','>','<','*','h'};


[FILE,path] = uigetfile('*.csv',...
   'Select One or More Files: *chose the same distance', ...
   'MultiSelect', 'on');


[Wfile,Wpath] = uigetfile('*.xlsx',...
    'Select the excel file containing manual data');


[WeightD,WeightS] = xlsread(convertCharsToStrings(Wfile));


WeightList = WeightS(2:length(WeightD)+1,1);


loop = length(FILE);


for i=1:loop


    File = FILE(1,i);


    fileID = fopen(convertCharsToStrings(File),'r');
C = textscan(fileID,'%q %*q %*d %f','Delimiter',',');


NAMEdat = C{1,1}(2,1);
NAMEdat = convertCharsToStrings(NAMEdat);
semiC = strfind(NAMEdat,';');
Parti{i} = extractAfter(NAMEdat,semiC(1,1));
Parti2{i} = extractBefore(Parti{i},semiC(1,2)-semiC(1,1));
DisplayN{i} = ['Swimmer ',num2str(i)]


Wfind{i} = find(strcmp([WeightList], [Parti2{i}]));


WeightData{i} = WeightD(Wfind{i},1);
SpeedData{i} = WeightD(Wfind{i},2);


stroke{i} = extractAfter(NAMEdat,semiC(1,3));
stroke2{i} = extractBefore(stroke{i},semiC(1,4)-semiC(1,3));


    name{i} = convertCharsToStrings(File);


    [SF{i},LOAD{i},MeanSpd{i},MeanFrc{i},MeanPwr{i},MaxSpd{i},MinSpd{i},Spd3{i},MeanRad{i}] =
Fun_Load_velocity_02Sep2019(File);


end


for i = 1:loop


    LOAD_sub = cell2mat(LOAD{i});
    MeanSpd_sub = cell2mat(MeanSpd{i});
    MeanFrc_sub = cell2mat(MeanFrc{i});
    MeanPwr_sub = cell2mat(MeanPwr{i});


     data1(1:length(LOAD_sub),i*2-1) = LOAD_sub';
     data1(1:length(MeanSpd_sub),i*2) = MeanSpd_sub';


    L = length(LOAD_sub);
```

```
    X{i} = [ones(length(LOAD_sub),1) LOAD_sub'];
    b{i} = X{i}\MeanSpd_sub';
    Y{i} = X{i} * b{i};

    X2{i} = [ones(length(MeanFrc_sub),1) MeanFrc_sub'];
    b2{i} = X2{i}\MeanSpd_sub';
    Y2{i} = X2{i} * b2{i};

    SIZE(i,:) = size(LOAD_sub);
    MaxLOAD(i,1) = max(LOAD_sub);

    clear LOAD_sub
    clear MeanSpd_sub
    clear MeanFrc_sub
    clear MeanPwr_sub

end

DataL = max(SIZE);
DataL = DataL(1,2);
data1 = zeros(DataL,loop);

for i = 1:loop

    LOAD_sub = cell2mat(LOAD{i});
    MeanSpd_sub = cell2mat(MeanSpd{i});
    SF_sub = cell2mat(SF{i});

     data1(1:length(LOAD_sub),i*3-2) = LOAD_sub';
     data1(1:length(MeanSpd_sub),i*3-1) = MeanSpd_sub';
     data1(1:length(MeanSpd_sub),i*3) = SF_sub';



    clear LOAD_sub
    clear MeanSpd_sub

end

LimLOAD = max(MaxLOAD);

for i = 1:loop

    LOAD_sub = cell2mat(LOAD{i});
    K{i}(1,1) = find(LOAD_sub==1);
    K{i}(1,2) = find(LOAD_sub==5);
    K{i}(1,3) = find(LOAD_sub==9);
    MeanSpd_sub(1,1) = cell2mat(MeanSpd{i}(1,K{i}(1,1)));
    MeanSpd_sub(1,2) = cell2mat(MeanSpd{i}(1,K{i}(1,2)));
    MeanSpd_sub(1,3) = cell2mat(MeanSpd{i}(1,K{i}(1,3)));
    MeanFrc_sub(1,1) = cell2mat(MeanFrc{i}(1,K{i}(1,1)));
    MeanFrc_sub(1,2) = cell2mat(MeanFrc{i}(1,K{i}(1,2)));
    MeanFrc_sub(1,3) = cell2mat(MeanFrc{i}(1,K{i}(1,3)));



    Xplot = X{i};
    bplot = b{i};
    Yplot = Xplot.*bplot(2,1)+bplot(1,1);
    bplot2 = b2{i};

    Full2(i,1) = -bplot2(1,1)./bplot2(2,1);
```

```
    SSE(i,1) = 0;
    SST(i,1) = 0;

    for j=1:length(LOAD_sub)

        SSE(i,1) = SSE(i,1) + (MeanSpd_sub(1,j)-(LOAD_sub(1,j).*bplot(2,1)+bplot(1,1))).^2;
        SST(i,1) = SST(i,1) + (MeanSpd_sub(1,j) - mean(MeanSpd_sub)).^2;

    end

    R2(i,1) = 1-(SSE(i,1)./SST(i,1));
    R2adj(i,1) = 1-((SSE(i,1)./(length(LOAD_sub)-2))./(SST(i,1)./(length(LOAD_sub)-1)));

    Lim = -bplot(1,1)./bplot(2,1);

    TimeLine = 0:0.01:Lim;
    Yplot2 = TimeLine.*bplot(2,1)+bplot(1,1);

    Full(i,1) = -bplot(1,1)./bplot(2,1);



    Marker = {'o','x','s','d','p','+','^','v','>','<','*','h'};
if (0<i) && (i<8)
       marker = Marker{1,1};
    elseif (7<i) && (i<15)
        marker = Marker{1,2};
    elseif (14<i) && (i<22)
        marker = Marker{1,3};
    elseif (21<i) && (i<29)
        marker = Marker{1,4};
    elseif (28<i) && (i<36)
        marker = Marker{1,5};
end

plot(LOAD_sub,MeanSpd_sub,marker,TimeLine,Yplot2,'LineWidth',1,'DisplayName',DisplayN{i})
xticks(0:1:50);
yticks(0:0.1:3.0);
title('Load Velocity curve')
xlabel('Load (kg)')
ylabel('Velocity (m/s)')
legend

set(gcf, 'Position',  [100, 100, 1200, 800])

hold on

data2(i,:) = horzcat(Full(i,1),b{i}(1,1),b{i}(2,1),...
    (b{i}(1,1)./2-bplot(1,1))./bplot(2,1),...
    (b{i}(1,1).*0.9-bplot(1,1))./bplot(2,1),...
    b{i}(1,1)./2,b{i}(1,1).*0.9,WeightData{i},Full(i,1)./WeightData{i}.*100,...
SpeedData{i},R2(i,1),R2adj(i,1));

end



TITLE1 = strings();
TITLE2 = {'L0','Vmax','Slope','Lopt','Ldec10%','vopt','vdec10%','Body mass',...
    'L0 (%bodyweight)','Race_maxV','R2','Adjusted_R2'};
```

```
for i=1:loop

    Tname = [Parti2{i}];
    Tname = convertCharsToStrings(Tname);

    TITLE1(1,i*3-2) = Tname;
    TITLE1(1,i*3-1) = stroke2{i};
    TITLE1(1,i*3) = 'SF'

    TNAME(i,1) = Tname;






end

for k=1:DataL

    TrialName{k,1} = ['Trial ',num2str(k)];

end

date = char(datetime);
Date = strrep(date,':','');

data1(data1==0)=NaN;




xlswrite(['Sprint_OUTPUT_',Date], TrialName, 'Overall','A2');
xlswrite(['Sprint_OUTPUT_',Date], TITLE1, 'Overall','B1');
xlswrite(['Sprint_OUTPUT_',Date], data1, 'Overall','B2');
xlswrite(['Sprint_OUTPUT_',Date], TNAME, 'Overall',['A',num2str(L+6)]);
xlswrite(['Sprint_OUTPUT_',Date], TITLE2, 'Overall',['B',num2str(L+5)]);
xlswrite(['Sprint_OUTPUT_',Date], data2, 'Overall',['B',num2str(L+6)]);
xlswritefig(gcf, ['Sprint_OUTPUT_',Date], 'Overall', ['A',num2str(L+6+loop+2)]);
```
