# Supplementary material for: Reliability of Load-Velocity Profiling in Front Crawl Swimming
Source: Front Physiol. 2020 Sep 23;11:574306. doi: 10.3389/fphys.2020.574306 (PMC7538691; doi:10.3389/fphys.2020.574306)
Supplement: Supplementary file 2 [file Data_Sheet_2.pdf]

Supplement 4. The MATLAB function for processing the data.

Olstad BH, Gonjo T, Njøs N, Abächerli K and Eriksrud O (2020)
Reliability of Load-Velocity Profiling in Front Crawl Swimming.
Front. Physiol. 11:574306. doi: 10.3389/fphys.2020.574306

```
function  [SF,LOAD,MeanSpd,MeanFrc,MeanPwr,MaxSpd,MinSpd,Spd3,MeanRad] =
Fun_Load_velocity_02Sep2019(FILE)

name = convertCharsToStrings(FILE);

file = readmatrix(name);



load1 = file(:,12);
load2 = file(:,13);

Reps = file(:,7);
Position = file(:,18);
Speed = file(:,19);
Acc = file(:,20);
Force = file(:,21);
Power = Speed .* Force;
sps = file(:,22);

sample = length(Reps);
No_Reps = Reps(sample,1);

REPS = zeros(sample,No_Reps);

Table_title = [];

for i=1:No_Reps

    check = find(Reps == i);

   LOAD{i} = load1(check(1,1)+1,1);

   POSITION{i} = Position(check(1,1):check(length(check)),1);
   SPEED{i} = Speed(check(1,1):check(length(check)),1);
   ACC{i} = Acc(check(1,1):check(length(check)),1);
   FORCE{i} = Force(check(1,1):check(length(check)),1);
   SPS{i} = sps(check(1,1):check(length(check)),1);
   POWER{i} = Power(check(1,1):check(length(check)),1);
   TIME{i} = (check(:,1)-check(1,1)) .* sps(2,1);

   Pstn_range{i}= POSITION{i}<15 & POSITION{i}>1;

   K{i} = find(Pstn_range{i});

   Bf{i} = K{i}(1,1);
   Bl{i} = K{i}(length(K{i}),1);



   Pstn{i} = POSITION{i}(Bf{i}:Bl{i},1);
   Spd{i} = SPEED{i}(Bf{i}:Bl{i},1);
   Accrlatn{i} = ACC{i}(Bf{i}:Bl{i},1);
   Frc{i} = FORCE{i}(Bf{i}:Bl{i},1);
%    SPS{i} = sps(Bf{i}:Bl{i},1);
   Pwr{i} = POWER{i}(Bf{i}:Bl{i},1);
   TIME{i} = [Bf{i}-Bf{i}:Bl{i}-Bf{i}]' .* sps(2,1);

   NoSpd{i} = Spd{i} - mean(Spd{i});

      [r{i},lags{i}] = xcorr(NoSpd{i}, 'coeff');

%       figure
```

```
      plot(lags{i}.*sps(2,1),r{i});

      [pk{i},lc{i}] = findpeaks(r{i},'MinPeakDistance',0.8./sps(2,1));

%       hold on
%       pks = plot(lags{i}(lc{i}).*sps(2,1),pk{i},'or');
%
%       hold off

EndP{i} = lc{i}(round(length(lc{i})./2),1);
BegP{i} = lc{i}(round(length(lc{i})./2)-3,1);
Cyclep{i} = lc{i}(round(length(lc{i})./2)-3:round(length(lc{i})./2),1);
CycleP{i} = Cyclep{i}-Cyclep{i}(1,1)+1;

SF{i} = 1./((EndP{i}-BegP{i}).*sps(2,1)./3);

   Pstn2{i} = Pstn{i}(BegP{i}:EndP{i},1);
   Spd2{i} = Spd{i}(BegP{i}:EndP{i},1);
   Accrlatn2{i} =  Accrlatn{i}(BegP{i}:EndP{i},1);
   Frc2{i} = Frc{i}(BegP{i}:EndP{i},1);
%    SPS{i} = sps(BegP{i}:EndP{i},1);
   Pwr2{i} = Pwr{i}(BegP{i}:EndP{i},1);
   TIME2{i} = [BegP{i}-BegP{i}:EndP{i}-BegP{i}]' .* sps(2,1);

   Rad{i} = asin(1./ Pstn2{i});
   Spd3{i} = Spd2{i}.*cos(Rad{i});
   Frc3{i} = Frc2{i}.*cos(Rad{i});
   Pwr3{i} = Pwr2{i}.*cos(Rad{i});

   MeanRad{i} = mean(Rad{i});

   MaxV{i}(1,1) = max(Spd3{i}(CycleP{i}(1,1):CycleP{i}(2,1)-1,1));
   MaxV{i}(1,2) = max(Spd3{i}(CycleP{i}(2,1):CycleP{i}(3,1)-1,1));
   MaxV{i}(1,3) = max(Spd3{i}(CycleP{i}(3,1):CycleP{i}(4,1)-1,1));
   MinV{i}(1,1) = min(Spd3{i}(CycleP{i}(1,1):CycleP{i}(2,1)-1,1));
   MinV{i}(1,2) = min(Spd3{i}(CycleP{i}(2,1):CycleP{i}(3,1)-1,1));
   MinV{i}(1,3) = min(Spd3{i}(CycleP{i}(3,1):CycleP{i}(4,1)-1,1));



   MeanSpd{i} = mean(Spd3{i});
   MaxSpd{i} = mean(MaxV{i});
   MinSpd{i} = mean(MinV{i});
   MeanFrc{i} = mean(Frc3{i});
   MeanPwr{i} = mean(Pwr3{i});



   if POSITION{i}(length(POSITION{i}),1) < 10
       SF{i} = [];
       LOAD{i}=[];
       MeanSpd{i}=[];
       MeanFrc{i} = [];
       MeanPwr{i} = [];
   end

clear check
end

end
```
