# Supplementary figures and images for: Reliability of Load-Velocity Profiling in Front Crawl Swimming
Source: Front Physiol. 2020 Sep 23;11:574306. doi: 10.3389/fphys.2020.574306 (PMC7538691; doi:10.3389/fphys.2020.574306)

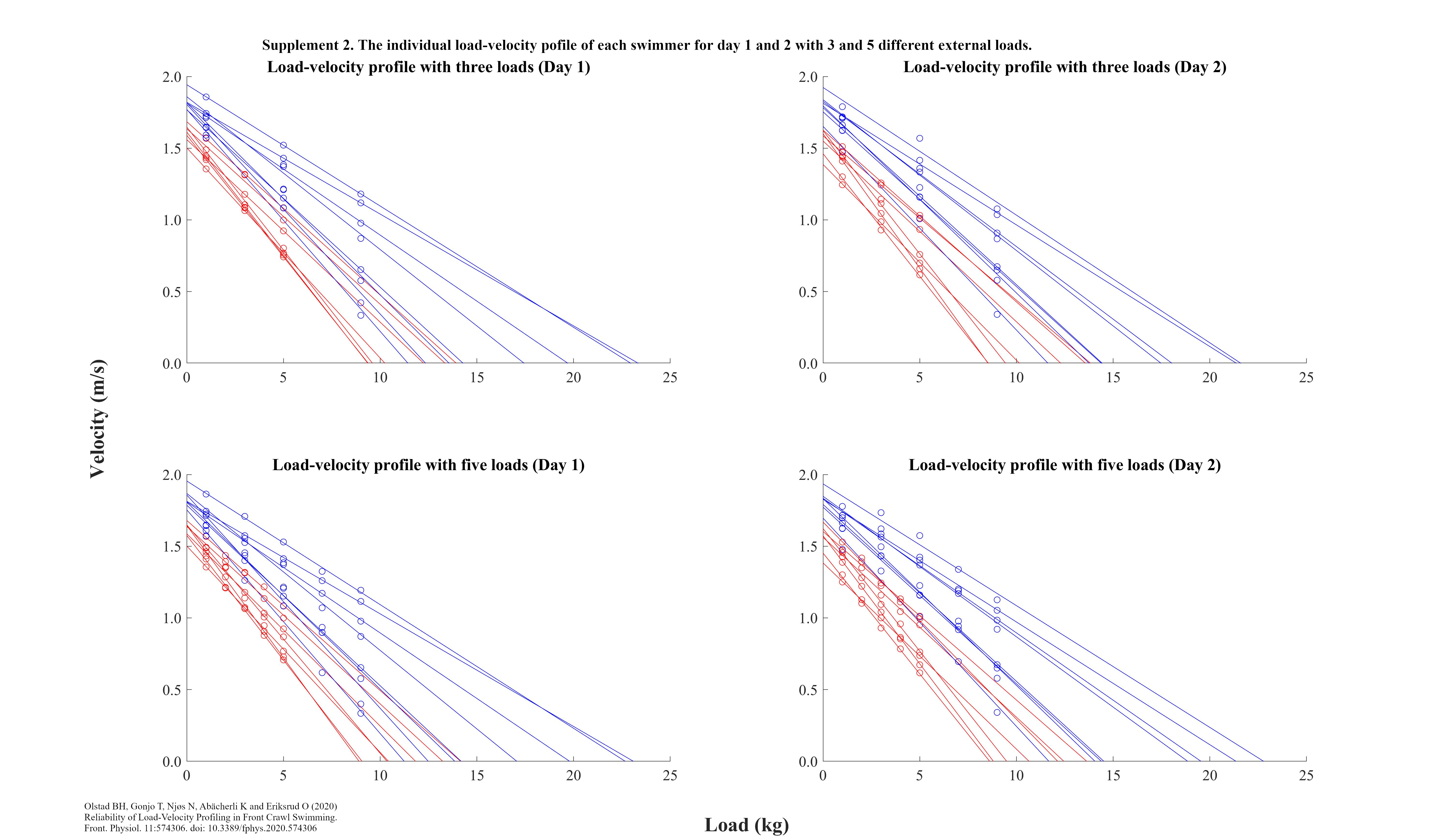

Supplement: Supplementary file 3 [file Image_1.jpg]
