# Supplementary material for: Reliability of Load-Velocity Profiling in Front Crawl Swimming
Source: Front Physiol. 2020 Sep 23;11:574306. doi: 10.3389/fphys.2020.574306 (PMC7538691; doi:10.3389/fphys.2020.574306)
Supplement: Supplementary file 4 [file Table_1.pdf]

Supplement 1. The real outcomes' measures for each subject with five and three different loads.

5 Loads

Subject	Gender	Day 1 L_0	Day 2 L_0	Day 1 rL_0	Day 2 rL_0	Day 1 V_0	Day 2 V_0	Day 1 S_{lv}	Day 2 S_{lv}	Day 1 R^2	Day 2 R^2	Day 1 50 m FC time	Day 2 50 m FC time
1	Male	22,67	22,78	26,45	26,99	1,96	1,93	-0,086	-0,085	0,996	0,953	22,84	22,73
2	Female	14,16	13,64	21,79	20,72	1,68	1,60	-0,119	-0,118	0,998	0,994	27,42	27,76
3	Male	23,09	21,35	29,31	27,41	1,81	1,83	-0,079	-0,086	0,999	0,981	23,94	24,34
4	Female	10,43	10,65	19,57	20,03	1,50	1,38	-0,144	-0,130	0,997	0,999	29,28	30,49
5	Female	9,06	8,62	13,21	12,52	1,58	1,45	-0,174	-0,169	0,998	0,997	29,87	30,55
6	Female	11,83	12,45	16,73	17,49	1,59	1,56	-0,134	-0,125	0,988	0,982	28,39	28,34
7	Male	13,86	14,39	19,06	19,90	1,81	1,77	-0,130	-0,123	0,986	0,997	25,86	25,88
8	Male	11,24	11,68	15,79	16,38	1,75	1,70	-0,156	-0,145	0,985	0,984	25,71	26,06
9	Female	13,24	12,13	18,33	17,00	1,64	1,67	-0,124	-0,137	0,976	0,991	27,31	27,86
10	Female	8,91	8,81	15,71	15,79	1,64	1,57	-0,185	-0,179	0,999	1,000	28,22	28,55
11	Female	10,35	9,49	16,80	15,41	1,65	1,62	-0,148	-0,159	0,994	0,998	26,64	26,82
12	Male	17,06	18,86	25,66	28,36	1,87	1,85	-0,109	-0,098	0,986	0,984	24,15	24,04
13	Male	14,19	14,52	20,86	21,35	1,79	1,79	-0,126	-0,123	0,998	0,999	25,75	25,14
14	Male	19,80	19,54	27,12	26,77	1,81	1,83	-0,091	-0,094	0,998	0,995	24,18	24,41
15	Male	12,49	14,06	18,31	20,62	1,86	1,83	-0,149	-0,130	0,955	0,970	24,88	25,11

3 Loads

Subject	Gender	Day 1 L_0	Day 2 L_0	Day 1 rL_0	Day 2 rL_0	Day 1 V_0	Day 2 V_0	Day 1 S_{lv}	Day 2 S_{lv}	Day 1 R^2	Day 2 R^2	Day 1 50 m FC time	Day 2 50 m FC time
1	Male	22,96	21,60	26,79	25,60	1,94	1,92	-0,085	-0,089	1,000	0,954	22,84	22,73
2	Female	13,94	13,84	21,44	21,03	1,69	1,59	-0,121	-0,115	0,999	1,000	27,42	27,76
3	Male	23,35	21,38	29,63	27,44	1,82	1,81	-0,078	-0,085	1,000	0,995	23,94	24,34
4	Female	10,25	10,16	19,23	19,09	1,50	1,39	-0,147	-0,137	1,000	0,999	29,28	30,49
5	Female	9,38	8,55	13,68	12,43	1,59	1,46	-0,170	-0,171	1,000	0,998	29,87	30,55
6	Female	12,25	12,29	17,33	17,27	1,56	1,55	-0,127	-0,126	1,000	0,991	28,39	28,34
7	Male	13,57	14,39	18,66	19,90	1,82	1,75	-0,134	-0,122	0,988	0,999	25,86	25,88
8	Male	11,44	11,63	16,07	16,31	1,77	1,65	-0,155	-0,142	0,986	0,990	25,71	26,06
9	Female	13,36	13,57	18,51	19,01	1,64	1,63	-0,122	-0,120	0,971	0,999	27,31	27,86
10	Female	9,36	8,53	16,52	15,29	1,61	1,60	-0,172	-0,188	1,000	1,000	28,22	28,55
11	Female	9,60	9,37	15,59	15,21	1,65	1,63	-0,172	-0,174	0,996	1,000	26,64	26,82
12	Male	17,44	17,48	26,23	26,28	1,86	1,84	-0,107	-0,105	0,986	0,991	24,15	24,04
13	Male	14,29	14,44	21,01	21,23	1,77	1,78	-0,124	-0,123	1,000	1,000	25,75	25,14
14	Male	19,71	18,62	27,00	25,50	1,82	1,84	-0,092	-0,099	0,998	0,998	24,18	24,41
15	Male	12,35	13,76	18,11	20,17	1,81	1,80	-0,146	-0,130	0,961	0,981	24,88	25,11

Olstad BH, Gonjo T, Njøs N, Abächerli K and Eriksrud O (2020)
Reliability of Load-Velocity Profiling in Front Crawl Swimming.
Front. Physiol. 11:574306. doi: 10.3389/fphys.2020.574306
